# Lumbar Disc Features Predictive of Favorable Response to Transforaminal Epidural Steroid Injection: a Comprehensive Review

**DOI:** 10.1007/s11916-026-01520-5

**Published:** 2026-07-20

**Authors:** Alexander Kervyn De Volkaersbeke, Sudeep Peddireddy, Gabriel Howard, Behnum Habibi

**Affiliations:** Temple University, 3401 N. Broad St., Philadelphia, PA 19140 USA

**Keywords:** Lumbar disc herniation, Transforaminal epidural steroid injection, Radicular pain, Nerve root compression, Clinical predictors, Pain management, Foraminal herniation, Extraforaminal herniation, Slump test, Treatment efficacy

## Abstract

**Background:**

Lumbar disc herniation (DH) is a prevalent condition, with transforaminal epidural steroid injections (TFESI) widely used to manage associated radicular pain. Despite its common use, the predictive value of various clinical and imaging characteristics, particularly the location of LDH, on TFESI outcomes remains unclear.

**Objectives:**

This review evaluates the influence of LDH location and other imaging or clinical predictors on TFESI efficacy, hypothesizing that more lateral herniations may be associated with better treatment outcomes.

**Study Design:**

A systematic review of 13 studies analyzing TFESI outcomes in patients with LDH was conducted.

**Setting:**

Ambulatory or outpatient setting.

**Methods, Patients, Intervention, Measurement:**

Articles included patients undergoing TFESI monotherapy for pain management due to lumbar DH. Studies assessing disc morphology, herniation location (central, paracentral, foraminal, extraforaminal), and/or nerve root compression were reviewed. Outcome measures included Visual Analog Scale (VAS), Oswestry Disability Index (ODI), and other validated pain/disability indices.

**Results:**

Three studies reported significantly better outcomes for lateral herniations (foraminal, extraforaminal), while six studies found no significant difference by location. Two studies suggested better outcomes in central herniations. Mild nerve compression, positive Slump test findings, and optimal TFESI technique correlated strongly with favorable outcomes. Severe nerve compression and technical challenges with more lateral herniations negatively impacted efficacy.

**Limitations:**

Heterogeneity in study design, outcome measures, and follow-up intervals limited generalizability. Many studies lacked control groups or long-term follow-up. Additionally, most of the included studies used particulate steroids.

**Conclusion:**

LDH location influences TFESI response, with mild compression and lateral herniations generally yielding favorable outcomes. However, technical proficiency and clinical predictors, such as positive Slump test, may be stronger determinants of success than herniation location alone. Further standardized research is required to optimize patient selection and elucidate the prognostic factors that contribute to TFESI treatment response.

## Introduction

Lumbar disc herniation is a very common condition with an estimated prevalence of 5.5% in the worldwide population and lifetime risk of 30% [1]. Although many cases remain asymptomatic, it is estimated that 1–3% of symptomatic cases are due to compression of the surrounding nerve structures [2]. Epidural steroid injections are commonly performed as an interventional treatment for radicular back pain due to disc herniation. Epidural steroid injections are often utilized after failure of more conservative treatments and prior to initiation of surgical management, although this varies greatly. There are three main approaches for epidural steroid injections: transforaminal, interlaminar, and caudal. Transforaminal epidural steroid injections (TFESI) are often chosen when targeted delivery of medication is a priority. A systematic review by Smith et al. reported that approximately 60% of patients with disc herniation experienced > 50% pain improvement one year after TFESI [3]. Despite the widespread use of TFESI, prognostic factors that predict favorable treatment response have remained unclear. It has been hypothesized that the site of disc herniation according to Wiltse classification (i.e. central, paracentral, subarticular, foraminal, extraforaminal) may influence treatment response to TFESI [4–7]. Wiltse et al. first described various anatomic zones of interest in relation to potential disc herniations in 1997, a classification which is still commonly used in clinical practice [8]. Moreover, it has been proposed that disc herniation in the lateral zones may cause more severe symptoms than central and paracentral zone herniations due to more direct compression of exiting nerve roots [9]. It is often assumed that lateral zone herniations respond more reliably to TFESI, given the more direct application of medication to the nerve root in comparison to interlaminar or caudal epidurals. However, to our knowledge, there is no systematic review of the current literature to evaluate the validity and nuance of this assumption. In this review, the authors seek to assess disc herniation location and other potential prognosticators of patient response to TFESI [10].

## Methods

Our study sourced articles from two of the largest publicly available medical databases including the PubMed and Embase libraries. These online databases are commonly used in literature review and medical research. Our search was performed on August 19th, 2024 for all-time publication data without geographic, primary language, or date screening. Search results must include the terms “Transforaminal” or “TFESI”, “Far-lateral disc”, “Disc herniation”, or “Sciatica” and “Injection”. Articles were then reviewed based on strict inclusion and exclusion criteria in both abstract/title review as well as full-text literature review for inclusion into the study. For inclusion, articles must be available with English translation and full text manuscript must be available. Articles must perform lumbar TFESI in the setting of disc herniation for the purposes of pain management. TFESI must be the primary intervention of the study with no alternative epidural approaches additionally supplied. Disc morphology as well as radiographic location (central, paracentral, subarticular, etc.) must be noted and analyzed for significance in relation to treatment response. Articles were excluded from the review if the inclusion criteria were not met and if any exclusionary criteria were present. Exclusionary criteria included non-human studies, case reports, cervical/thoracic spine TFESI, presence of alternative/competing lumbar interventions, and TFESI for any reason other than for the purposes of pain management. Articles which involved dual or competing therapies to treatment arms (caudal, interlaminar epidural steroids, etc.) were also removed to more clearly isolate treatment response to TFESI alone. Lumbar disc herniation and radiographic location with respect to laterality must be identified, articles reporting morphology alone (bulge, herniation, extrusion etc.) or presence of nerve impingement alone were excluded. There were 793 articles which resulted after the initial search with 239 duplicates which were removed. Three blinded reviewers performed title and abstract review of 554 reports for eligibility. Any conflicts that arose after blinded review were resolved with unblinded discussion among all three reviewers. Twenty-four reports met the inclusion and exclusion criteria of abstract/title review and were sought for full text retrieval. Six articles were unavailable for retrieval, three articles were removed as there were insufficient data regarding lumbar disc location, and one final article was removed for providing additional treatment (discectomy) to the intervention group.

## Results

Across the body of evidence, a consistent narrative emerges: transforaminal epidural steroid injection (TFESI) generally provides meaningful short to medium-term relief of radicular pain from lumbar disc herniation, but the magnitude and durability of benefit depend on an interplay between anatomy, pathology severity, and procedural technique rather than any single factor alone.

At a foundational level, multiple studies converge on the same baseline observation: pain and disability improve after TFESI across a wide spectrum of disc herniation types. For example, Serifoglu et al.^14^ demonstrated significant reductions in both VAS and ODI scores at three months across different lumbar levels, while Kim et al.^5^ similarly showed meaningful improvements at 12 weeks in both central and far lateral disc herniations without a significant difference between groups. Together, these findings reinforce that TFESI has broad efficacy independent of level or basic morphology.

When shifting from general effectiveness to predictors of who benefits most from TFESIs, severity of nerve root compression emerges as one of the most consistent determinants of outcome. Both Veljanovski et al.^13^ and Bogduk and Gahreman^2^⁰ found that low-grade nerve compression was associated with significantly better pain reduction, whereas higher grades of compression predicted diminished response. This pattern supports the idea that TFESI is most effective when inflammation, rather than fixed mechanical compression, is the dominant pain driver.

The role of herniation location is more complex and sometimes contradictory, but still informative. Veljanovski et al.^13^ and Lee et al.^4^ reported more favorable outcomes in foraminal or extraforaminal herniations, suggesting that these locations may be particularly amenable to targeted steroid delivery. In contrast, the large cohort analyzed by Guclu et al.^21^ found greater pain relief in paramedian herniations compared to foraminal ones. Meanwhile, Kwak et al.^1^⁹ observed no significant long-term differences across herniation locations. Taken together, these findings suggest that location alone is not determinative but instead interacts with factors like nerve compression severity and technical access to the affected nerve root.

In comparison, disc morphology (e.g., protrusion vs. extrusion) appears to play a relatively minor and inconsistent role. Tecer et al.^1^⁷ found that TFESI improved pain regardless of morphology or location, while Kwak et al.^1^⁹ noted only indirect effects (such as differences in repeat injection rates rather than clear efficacy differences). Even when morphology showed some associations such as improved outcomes with sequestration patterns in Lechman et al.⁶, these findings were not consistently replicated across studies. This suggests that structural classification of the disc is less predictive than its functional impact on neural structures.

Beyond static imaging features, several studies highlight the importance of clinical and radiologic nuance. For instance, Ekedahl et al.^1^⁶ identified a positive slump test and high-grade subarticular stenosis as predictors of short-term improvement, while Tecer et al.^1^⁷ found that high-intensity zones and nerve root impingement correlated with better outcomes at early follow-up. However, these predictors are not universally consistent, reinforcing that no single MRI or exam finding reliably forecasts response across all patients.

Perhaps most compellingly, the literature points to procedural technique as a critical, and sometimes dominant, factor. Jung et al.^1^⁸ demonstrated that needle tip proximity to the affected nerve root, extra-epineural contrast spread, and proximal flow patterns were strongly associated with favorable outcomes, with needle positioning emerging as the most important predictor. This suggests that even in the presence of less favorable anatomy, precise delivery of medication can significantly influence clinical success.

Finally, several studies underscore the practical limitations and variability of TFESI in real-world settings. For example, Kim et al.^5^ reported technical failure in a substantial proportion of far lateral disc herniation cases due to intolerable procedural pain, and Evran and Katar noted that some patients ultimately required surgery despite initial treatment. These findings emphasize that while TFESI is broadly effective, it is not universally feasible or sufficient as a standalone intervention.

In synthesis, TFESI can be understood as a generally effective but selectively optimized therapy: most patients experience meaningful relief, but the best outcomes are seen in those with less severe nerve compression, favorable or accessible anatomy, and accurately targeted injections, with procedural precision potentially outweighing many traditional imaging predictors.

See Table 1 for a full summary of all resulted articles.

**Table 1:** Synthesized results of all studies

Title	Population	Intervention	Key Independent Variables Assessed	Outcome
Transforaminal Epidural Injection for Far Lateral Lumbar Disc Herniations: An Alternative to Surgery or Just a Delay? (Serifoglu)	42 patients w/ lateral DHs subdivided into level of path. (L3/4 vs L4/5 vs L5/S1)	TFESI w/ measured changes in VAS/ODI at 3 months	Far-lateral disc herniation only	TFESI results in improved ODI and VAS at 3 months for lateral disc herniations
Clinical Effectiveness of Single Lumbar Periradicular Infiltration in Patients with Sciatica. (Veljanovski)	166 patients w/ subgroups based on degree nerve compression, DH location	TFESI w/ measured changes in VAS at 6 months	Degree of nerve compression and location of DH	TFESI improved VAS in patients with low-grade root compression with DH particularly in posterolateral and extraforaminal zones without high grade nerve compression at 6 months
The efficacy of transforaminal epidural steroid injection by the conventional technique in far-lateral herniation of lumbar disc. (Kim)	85 patients. (15 with far-lateral DH and 70 with central DH)	TFESI w/ VAS/ODI score changes at 12w	Location of DH. (FLDH vs CDH)	TFESI performed for FLDH and CDH with both groups showing statistical significance from baseline but not from each other
Evaluation of the Effectiveness of Transforaminal Epidural Steroid Injection in Far Lateral Lumbar Disc Herniations (Evran and Katar)	37 patients with single-level far lateral disc herniation seen on lumbar MRI	TFESI w/ VAS and ODI pre-injection and post-injection at 3 weeks, 3 months and 6 months	Far-lateral disc herniation only	FLDH more likely to be in “very best” response group while CDH were more likely to be in the “very worse” group after TFESI
MR-based outcome predictors of lumbar transforaminal epidural steroid injection for lumbar radiculopathy caused by herniated intervertebral disc. (Lee and Choi)	149 patients who underwent TFESI – 87 with “very worst outcome”, 62 with “very best outcome.”	TFESI w/ VAS and 5-point self-satisfaction scale from 2w through 2y follow up	Age, DH location, nerve root compression grading, disc degeneration	Foraminal/extraforaminal predicts best outcome compared to central/subarticular; Among older people (60 m +) sig more in best outcome compared to worst outcome
Relationship of specific MRI findings to treatment outcomes in patients receiving transforaminal epidural steroid injections. (Lechman)	156 patients with disc herniation identified on MRI received TFESI and completed improvement based questionnaires at 1 month post-injection	TFESI completed with NRS and Patients Global Impression of Change (PGIC) at 1 month follow-up	Disc morphology and laterality, nerve root compression grading, and burden of central canal stenosis	Patients with disc protrusion plus sequestration, paracentral nerve root deviation or severe foraminal nerve root compression, and foraminal/extraforaminal disc herniation location reported the highest pain reduction in NRS following TFESI
Three-week results of transforaminal epidural steroid injection in patients with chronic unilateral low back related leg pain: The relation to MRI findings and clinical features. (Ekedahl)	100 patients with radicular low back pain and related MRI findings underwent TFESI and assessed at 3 weeks post-injection	TFESI completed with ODI, VAS, and Slump test and neurologic assessment at 3 wks	Disc Herniation location, grade of nerve root compression, clinical neurologic deficit, presence of slump test	Presence of positive slump test had the greatest predictive value for positive TFESI response while MRI findings failed to predict response
Role of Magnetic Resonance Imaging in Ascertaining the Success of Transforaminal Epidural Steroid Injection for Lumbar Radicular Pain (Tecer)	59 patients who had lumbar radicular pain with recent lumbar MRI	TFESI with VAS monitoring at 2 weeks and 3 months post-injection	Disc herniation type, location, presence of high intensity zone (HIZ), and degree of nerve root impingement	TFESI improved radicular pain symptoms irrespective of disc herniation location and morphology with largest improvement in patients with HIZ at 2 weeks and root impingement at 3 months
The use of magnetic resonance imaging to predict the clinical outcome of non-surgical treatment for lumbar intervertebral disc herniation. (Choi)	68 patients with lumbosacral disc herniations on CT or MRI were treated with TFESI	TFESI with subjective responses of 0–4 (poor, fair, good, very good, excellent) and VAS scoring tracked at variable follow up periods	Disc herniation morphology, hydration, location of herniation, grade of nerve compression	Location of disc herniation and grade of nerve root compression were closely associated with better response to TFESI, but not hydration, type or size of herniation
The Prognostic Value of Enhanced-MRI and Fluoroscopic Factors for Predicting the Effects of Transforaminal Steroid Injections on Lumbosacral Radiating Pain. (Jung)	51 patients with radicular LBP received TFESI and underwent retrospective analysis as “favorable” and “unfavorable” groups	TFESI w/ VAS scoring tracked from pre-injection to 2wk post-injection	Approach of injection, proportion of proximal contrast flow, location and disc morphology, and contrast dispersion patterns	TFESI approach to targeted nerve root is the most important factor in predicting response to TFESI
Predictors of a favorable response to transforaminal injection of steroids in patients with lumbar radicular pain due to disc herniation. (Bogduk/Gahreman)	71 patients w/ DH further divided into pathology level, DH location and grade of nerve root compression	TFESI w/ VAS scoring tracked from pre-injection to 4wk post-injection	Paramedian and Foraminal DH	Low grade compression was the only statistically significant factor in predicting TFESI success response
Transforaminal Epidural Steroid Injection in the Treatment of Pain in Foraminal and Paramedian Lumbar Disc Herniations. (Deniz)	1632 patients (1262 with paramedian DH and 370 with foraminal DH) underwent a single TFESI w/ 12wk follow up	TFESI w/ VAS scoring tracked from pre-injection to 12wk post-injection	Paramedian and Foraminal DH	TFESI significantly reduced pain for both types of herniations, however there was greater relief for those with paramedian DH compared to foraminal DH
Long-term outcomes of transforaminal epidural steroid injection in patients with lumbosacral radicular pain according to the location, type, and size of herniated lumbar disc. (Kwak)	160 patients (HLD only) with subgroup analysis by pathology level, DH location, protrusion vs extrusion and disc size	TFESI with presence of radicular pain, NRS rating of pain, medication usage as well as utilization of repeated TFESI assessed at 4yrs post injection	DH location, pathology level (L4/5 vs L5/S1), disc morphology (protrusion vs extrusion) and disc size	No difference in quality outcome metrics such as radicular pain, NRS rating of pain, and medication usage between paracentral vs foraminal DH at 4y

*Table 1 represents a full summary of all resulted articles

## Discussion

Our review included 13 studies which each assessed TFESI response with respect to independently measured MRI characteristics, exam findings and outcome measures. The primary focus of our review was assessment of DH location and TFESI response with secondary outcomes including treatment response to other reported variables. Most studies utilized either the Visual Analog Scale (VAS), Oswestry Disability Index (ODI), Numeric Rating Scale (NRS), Patient's Global Impression of Change (PGIC) or other validated tools to evaluate response to TFESI procedure. VAS and NRS are relatively simple self-reporting tools which utilize visual facial representations and numeric values for subjective pain measurement respectively [11]. PGIC is slightly more nuanced, aimed at detecting subjectively significant improvement in symptoms, while ODI is the most robust by assessing various domains of lifestyle including sleeping, standing, sitting, social life and other important factors [12, 13]. Articles included in this review utilized a variety of each of these outcome measures and at times found varying degrees of significance for similar cohorts. Studies varied in design with large variances in follow up interval, pain assessment scales, TFESI technique, medication selection as well as pre-procedural and post-procedural assessments. Despite lack of uniformity, there were emergent consistencies present in regards to DH characteristics and response to TFESI.

### Effectiveness of TFESI by DH Location

Three of the 13 included studies showed statistically significant improvement in TFESI response in favor of lateral disc herniations (LDH) cohorts as compared to more centralized anatomic zones of DH. Veljanovski et al., Lee et al., and Lechman et al., showed statistically significant pain relief in TFESI cohorts that had lateral or far lateral disc herniations compared to control cohorts that had more central herniations [4, 6, 14]. Veljanovski et al. and Lee et al. represented this difference through significant differences in VAS measures pre- and post-TFESI procedure across the two groups. Lechman et al., represented this difference through significant difference in NRS and PGIC scores of lateral/far lateral DH cohorts as compared to centralized DH cohorts. Of note, Veljanovski et al. also included non-DH pathology such as central canal stenosis in their study design; however, no statistically significant findings of improvement were seen in these alternative pathologies. Two studies also suggested significant improvements in pain measures among a far lateral disc herniation cohort. However, they were limited due to their lack of control comparisons across different anatomic zones. The studies conducted by Serifoglu et al., and Evran et al., showed significant improvements in both VAS and ODI measures post-procedure in their FLDH cohorts. Other criteria of evaluation, including level of pathology such as L4/5 vertebrae vs L5/S1, were not found to have any significant effect on results [10, 15].

There were six studies among the 13 that suggested a lack of significant difference between cohorts based on anatomic location of disc herniation. The studies conducted by Kim et al., Ekedahl et al., Tecer et al., Choi et al., Jung et al., and Kwak et al. all yielded findings that suggest a lack of significant difference between cohorts based on anatomic location of disc herniation [5, 7, 16–19]. All the aforementioned studies evaluated injections at 1–3 months post injection, with the exception of Kwak et al., which analyzed their cohort a minimum of 4 years after injection. Most of the studies analyzed changes in VAS score, whereas others such as Kwak et al., used NRS scoring. Ekedahl et al., also used the positive straight leg raise/slump test as a positive outcome predictor in conjunction with VAS scores. Kim et al., and Ekedahl et al., also utilized ODI scores in conjunction with VAS. The findings of Kwak et al., suggested that the only variable to achieve statistical significance in predicting outcome was whether disc herniations were protruded (better outcomes) vs extruded. Herniation location did not achieve statistical significance in that study. However, it is worth noting that foraminal/extraforaminal disc herniations did show reductions in NRS scoring 4 years post-procedure. Choi et al. draws mixed conclusions. They studied multiple variables including location of herniation, morphology, degree of nerve root compression and hydration. There was an association with better outcome post-TFESI in the more lateral DH zones, however this was due to a 1/1 positive response in the only patient that had an extraforaminal disc herniation. All 6 subarticular DH patients were non-responders i.e. did not have sufficient pain relief post-TFESI. This data remains limited with low statistical power.

There were two studies that suggested that more central herniations respond more reliably to TFESI, by demonstrating significant superiority in pain relief amongst central/paracentral/subarticular DH cohorts as compared to the lateral herniation cohorts. Ghahreman and Bogduk and Guclu et al. both utilized TFESI injections amongst patient cohorts divided by herniation location [20, 21]. Both found that VAS scores 1–3 months post injections were more reduced in paracentral/paramedian disc herniations as compared to more lateral herniations. Ghahreman and Bogduk further stratified their cohort by level of disc pathology as well as grade of nerve root compression and found that the only factor that achieved statistical significance was low grade nerve root compression (better outcomes) as compared to high grade compression (worse outcomes). However, there was a non-significant trend toward paramedian herniations having better outcomes than lateral herniations. In a similar finding, Guclu et al. observed that only their paramedian herniation cohort achieved statistical significance (p < 0.05) in the reduction of their VAS scores post injection. It is worth noting that both groups (paramedian and foraminal DH) showed improvement from pre- to post-TFESI VAS scoring.

### Nerve Root Compression and TFESI Response

The degree of nerve compression was an independent variable that was assessed in a number of studies. Veljanovski et al. and Bogduk and Gahreman independently concluded that low-grade nerve compression predicts better TFESI outcomes. Conversely, high-grade compression, as seen in Choi et al., correlated with poorer responses. Moreover, Lechman et al. and Choi et al. both relay similar findings that greater nerve root compression is correlated with less efficacious treatment response to TFESI. Additionally, Guclu et al. noted paramedian disc herniations showed greater pain relief than foraminal disc herniations after TFESI, again suggesting a correlation between location of herniation and severity of symptoms. These findings confirm the notion that greater nerve compression is associated with more severe symptoms, suggesting reduced efficacy of TFESI in managing pain in cases of severe compression.

### Clinical Indicators, Steroid type and Technical Considerations

There are several notable clinical findings in these studies that warrant further investigation. One such finding by Ekedahl et al. is that a positive Slump test was a reliable prognosticator of TFESI treatment response. This finding is highlighted by the fact that MRI imaging findings did not carry the same predictive value in this study. This is a useful and cost-effective prognosticator for TFESI treatment response. Kim et al. found a high technical failure rate with TFESI in far lateral disc herniations and 60% of the cohort had to abort the procedure due to pain during the needle insertion process. The need for technical skill is corroborated by Jung et al. who found that factors such as proximity of needle tip to targeted nerve root, extra-epineural distribution of contrast pattern, and high proportion of proximal contrast flow pattern were associated with favorable TFESI response. Additionally, it is interesting to note that a large majority of the included studies used particulate steroids, which is a notable deviation from the recommendation of most pain societies. Interestingly, the one study that used non-particulate steroids, namely Guclu et al., found that paramedian disc herniations had greater TFESI response than foraminal disc herniations.

## Conclusion

The aim of this systematic review was to determine the relationship between anatomic location of lumbar disc herniations and treatment response in transforaminal epidural steroid injections. It was hypothesized that more lateral (i.e. foraminal/extraforaminal zones of disc herniation lateral to the subarticular zone) herniations may predict more successful outcomes in pain reduction after monotherapy with TFESI procedure. Among studies included in our review, three supported the proposed hypothesis with adequate comparison to central herniations, with two additional articles suggesting similar findings in isolated cohorts of lateral disc herniations alone. Six of 13 articles maintained neutral findings of insignificant difference in improvement based on anatomic zone, and the remaining two papers suggested a contradictory finding of greater improvement in centralized/median zones of disc herniation. Each study described used unique study designs which are likely responsible for the discrepancies reported here. Despite the lack of unanimity in the available literature, some conclusions can be reasonably made. We believe DH location is a significant factor in predicting typical response to TFESI. Very centralized DH or disc bulges are often in the worst response groups with less favorable responses than far lateral and paracentral DH. Paracentral DH responses are variable, with best responding groups often demonstrating disco-radicular contact or mild nerve root compression. Likewise, foraminal DH response to TFESI is varied, although the best responding groups were associated with mild nerve root compression and worst responding groups with severe nerve root compression. Our review indicates that laterality of disc herniation may be associated with, but not directly correlated with response to TFESI. Other factors are more directly correlated with favorable TFESI response such as mild nerve root compression, TFESI technique, and evidence of Slump test on exam. Based on our review, a patient with paramedian or foraminal DH with mild nerve root compression on MRI and positive Slump test on physical exam who undergoes TFESI with ideal needle placement may expect the best outcomes.

## Limitations

This systematic review is limited by substantial heterogeneity across included studies, including variation in study design, outcome measures (VAS, ODI, NRS, PGIC), follow-up intervals, and TFESI techniques, which constrain direct comparability and generalizability of findings. Many studies lacked control groups or long-term follow-up, and several were underpowered for subgroup analyses, particularly by herniation location. Additionally, the predominance of particulate steroid use across studies may limit applicability to current practice patterns.

## Key References

- Lee JW, Choi SW, Park SH, et al. MR-based outcome predictors of lumbar transforaminal epidural steroid injection for lumbar radiculopathy caused by herniated intervertebral disc. *Eur Radiol*. 2013;23:205–211. 10.1007/s00330-012-2566-3.
  - ○ All three of the above references showed statistically significant pain relief in TFESI cohorts that had lateral or far lateral disc herniations compared to control cohorts that had more central herniations, in keeping with the initial hypothesis/aim of investigation of the review.
- Lechmann M, Rosskopf A, Ehrmann C, et al. Relationship of specific MRI findings to treatment outcomes in patients receiving transforaminal epidural steroid injections. *Skeletal Radiol*. 2016;45:1677–1685. 10.1007/s00256-016-2487-3.
  - ○ Lechman et al., represented this difference through significant difference in NRS and PGIC scores of lateral/far lateral DH cohorts as compared to centralized DH cohorts.
- Veljanovski D, Panev SD, Kostova M, et al. Clinical effectiveness of single lumbar periradicular infiltration in patients with sciatica. *Pril (Makedon Akad Nauk Umet Odd Med Nauki)*. 2023;44(2):149-156. 10.2478/prilozi-2023-0034.
  - ○ Veljanovski et al. and Lee et al. represented this difference through significant differences in VAS measures pre- and post-TFESI procedure across the two groups. Lee et al. found that herniations in the foraminal or extraforaminal zones were associated with better outcomes, while other factors like disc degeneration or nerve root compression were not significant predictors. Veljanovski et al. found the greatest improvements in VAS scores six months post-procedure in patients with extraforaminal disc herniation and those without high grade nerve compression.

## Data Availability

Data is provided within the manuscript as outlined in the References section at the end of the manuscript.
